# Spatio-Temporal Variation in Contrasting Effects of Resident Vegetation on Establishment, Growth and Reproduction of Dry Grassland Plants: Implications for Seed Addition Experiments

**DOI:** 10.1371/journal.pone.0065879

**Published:** 2013-06-05

**Authors:** Jana Knappová, Michal Knapp, Zuzana Münzbergová

**Affiliations:** 1 Department of Botany, Faculty of Science, Charles University in Prague, Prague, Czech Republic; 2 Institute of Botany, Academy of Sciences of the Czech Republic, Průhonice, Czech Republic; 3 Department of Ecology, Faculty of Environmental Sciences, Czech University of Life Sciences Prague, Prague, Czech Republic; Jyväskylä University, Finland

## Abstract

Successful establishment of plants is limited by both biotic and abiotic conditions and their interactions. Seedling establishment is also used as a direct measure of habitat suitability, but transient changes in vegetation might provide windows of opportunity allowing plant species to colonize sites which otherwise appear unsuitable. We aimed to study spatio-temporal variability in the effects of resident vegetation on establishment, growth and reproduction of dry grassland species in abandoned arable fields representing potentially suitable habitats. Seeds were sown in disturbed (bare of vegetation and roots) and undisturbed plots in three fields abandoned in the last 20 years. To assess the effects of temporal variation on plant establishment, we initiated our experiments in two years (2007 and 2008). Seventeen out of the 35 sown species flowered within two years after sowing, while three species completely failed to become established. The vegetation in the undisturbed plots facilitated seedling establishment only in the year with low spring precipitation, and the effect did not hold for all species. In contrast, growth and flowering rate were consistently much greater in the disturbed plots, but the effect size differed between the fields and years of sowing. We show that colonization is more successful when site opening by disturbance coincide with other suitable conditions such as weather or soil characteristics. Seasonal variability involved in our study emphasizes the necessity of temporal replication of sowing experiments. Studies assessing habitat suitability by seed sowing should either involve both vegetation removal treatments and untreated plots or follow the gradient of vegetation cover. We strongly recommend following the numbers of established individuals, their sizes and reproductive success when assessing habitat suitability by seed sowing since one can gain completely different results in different phases of plant life cycle.

## Introduction

Seedling establishment poses a core restriction on the colonization of new habitats and largely determines the viability and structure of plant populations and communities [1]. Successful establishment is limited by both biotic and abiotic conditions and their interactions [2]. It is clear that seedling establishment can vary greatly in space and time together with environmental conditions [3]. Spatial variability in environmental conditions or consequences of direct experimental manipulation has been widely studied with respect to seedling establishment [4]–[6]. In contrast, temporal environmental changes and their effect on establishment have received relatively less experimental attention although theory suggests their importance for colonization and community assembly [7], [8].

It has been repeatedly shown that resident plants compete with emerging seedlings [5], [9]–[11]. The resident vegetation, however, could also facilitate the establishment of other species [12], 13. The relative importance of competition and facilitation differs greatly among investigated species and environments [14], [15]. Facilitating effects may turn into competitive interactions along with changes in limiting environmental factors, such as moisture or temperature [16], [17]. As a result, similar vegetation might enhance seedling establishment at some sites and restrict at other sites. More importantly, windows of opportunity opened for establishment by disturbance might remain unexploited in one year and fully utilized in another, depending on weather [18], [19]. However, temporal repetition of field experiments is still surprisingly rare [20]. Interactions of resident vegetation and establishing seedlings may switch between competition and facilitation also as plants transition to different life-history stages. For example, resident vegetation may provide suitable microclimatic conditions for germination, but at the same time, it may reduce the growth and/or survival of germinated seedlings [21]–[23]. Therefore, the patterns observed in the early stages of plant development may not always correspond to those observed in later developmental stages [4].

Seedling establishment is often used as a direct measure of habitat suitability [24], [25]. Site occupancy seems to be a less proper measure as species might be absent from otherwise suitable sites due to their poor dispersal abilities. Successful establishment after experimental sowing (or transplantation) implies habitat suitability of previously unoccupied site [26]. Nonetheless, seed sowing experiments addressing habitat limitation often do not consider above mentioned complex effects of resident vegetation on seedling establishment. Transient changes in vegetation might provide windows of opportunity allowing plant species to colonize sites which otherwise appear unsuitable [27]. We therefore wonder if resident vegetation and its spatio-temporal changes affect establishment, growth and reproduction of colonizing plant species. To achieve this goal, we performed complex seed sowing experiment with multiple plant species typical for central-European dry grasslands. In one experimental design, we combined manipulation of vegetation cover, seasonal variability induced by two years when the experiment was initiated and spatial variability given by three experimental sites varying in productivity and water availability. The experiment was performed in abandoned fields which are perceived as alternative habitats for species from declining grasslands [28], [29]. Indeed, many species are able to spontaneously colonise abandoned fields [30]–[32] and at certain circumstances, abandoned fields could be even more species rich than overgrowing grasslands [33]. However, many other species are absent from communities that develop in abandoned fields and why this occurs is an important question [32], [34], [35].

The main aim of our study was (i) to determine the ability of a range of dry grassland species to become established after sowing on abandoned fields, (ii) to assess the general effect of resident vegetation on species establishment, growth and reproduction and (iii) to determine role of seasonal variability on plant response to resident vegetation. To identify how the effect of vegetation changes depends on species traits and abiotic factors, we asked the following, more specific questions: (iv) Which plant traits can explain species-specific performance under different conditions? (v) Which site conditions modify the effect of vegetation on plant performance? We hypothesize that species that are taller or have larger seeds are better adapted to withstand shading from vegetation and thus be less sensitive to surrounding vegetation. We also expect stronger suppressing effect of vegetation on sown species on sites which are richer in nutrients due to greater vigour of vegetation and stronger shading.

## Methods

No specific permits were required for entering experimental localities and other sites used for collecting material for the experiment as they were not privately owned or protected in any way. The study involved several endangered or protected species. For manipulating seed material of these species, JK received approval from the Ministry of Environment of the Czech Republic.

### Study sites

The field seed sowing experiment was performed in the north-western part of the Czech Republic. The long-term average temperature (mean annual temperature over period 1961–1990) in the region is 7.7°C, and long-term normal precipitation is 612 mm [36]. Seed sowing took place in autumn 2007 and 2008, and most plants therefore germinated in spring 2008 and 2009, respectively. In 2008, May and June were abnormally dry, whereas in 2009, monthly precipitation levels from May to July were above the long-term normal (see Appendix S1 in Supporting Information).

The region is characterised by abundant fragments of species-rich calcareous dry grasslands (alliance *Bromion erecti*) surrounded mainly by arable fields. Some of these fields have been abandoned in the last two decades, and they are currently undergoing secondary succession. Most of the grassland sites are currently not managed and the succession at these sites will eventually lead to communities dominated by shrubs and then hornbeam forests. Similarly, most of the abandoned fields are currently not managed. The rate of shrub encroachment at these sites is, however, very slow (more than 50 years) due to dry climate and shallow soils in the region. From our observations, it is thus reasonable to assume that the succession on the abandoned fields will lead to grass dominated communities in the next few decades.

Three fields abandoned in the last 20 years were chosen for the seed sowing experiment. All selected fields were overgrown with grasses and ruderal herbaceous vegetation. The fields are dominated by few ruderal species such as *Cirsium arvense*, *Daucus carota*, *Melilotus officinalis*, *M. alba*, etc. (see Appendix S2 in Supporting Information), and the vegetation of the fields is very homogenous. Thus, although we did not record detailed composition of resident vegetation prior to sowing and disturbing the vegetation (and could not obtain the data afterwards as one experimental field was re-ploughed (just after final autumn census of experimental plots), it is reasonable to assume that there were no major differences in vegetation composition between experimental plots within each field.

To characterise the whole fields, we assessed site productivity as biomass at the end of the growing season representing overall level of competition from the neighbouring plants for the germinating seedlings. All aboveground biomass was harvested in four squares 0.5×0.5 m per field, distributed in proximity of experimental blocks (see below). The variation between productivity estimates in these 4 squares was very low due to the homogeneity of the vegetation and the data thus represent the fields well.

Due to political and socio-economical situation in former Czechoslovakia before 1989 (land expropriation), the ownership of the fields was still unclear at the time of experiment. For the same reason, it was also not possible to assess the exact time since abandonment, since no formal documentation exists about the former management. However, according to personal communication with land managers we assume all the fields being approximately of the same age (i.e. abandoned 15–20 years ago).

To evaluate differences in habitat conditions between the three fields, data on soil properties were collected for each field. Six soil samples per block (see below for block definition) were taken in autumn 2007 and C (total, carbonate and organic), N, P, K, Ca and Mg concentrations and pH (in both water and KCl solutions) were analyzed in the laboratory (for methods, see e.g. [37]). Additionally, we took 6 soil cores (100 cm^3^ each) per block to assess the maximum water holding capacity (WHC [3]). The three fields vary in most of the measured soil characteristics. Because we only have 3 localities, it is not possible to test the effects of characteristics of the localities on the results. We thus use these data only to describe the localities. The strongest difference between the three fields seems to be given by P concentrations (high in one field and low in two remaining fields; Appendix S2) the higher being manifested by higher standing biomass and also associated with lower C, N, K, Ca and Mg concentrations. The second most important difference seems to be WHC (inversely correlated with pH), similarly being high in one field and low in two other fields. Although there were differences also in the other soil characteristics (and likely in other unmeasured factors as well), we assume that P concentrations and WHC have the highest importance for plant responses. Simplified, we can distinguish the three fields as (1) nutrient rich with low WHC, (2) nutrient poor with low WHC and (3) nutrient poor with high WHC.

### Experimental set-up

In each experimental field, three blocks each comprising of two disturbed and two undisturbed plots were established. One disturbed and one undisturbed plot in each block were established and sown at the end of November 2007, and the remaining two plots (one disturbed and one undisturbed) were established and sown at the end of November 2008. In the disturbed plots, the soil was trenched immediately prior to seed sowing to a depth of approximately 0.3 m, and turfs and roots were removed to minimise the resprouting of original vegetation from vegetative organs. In the undisturbed plots, no alterations were made prior to seed sowing. No further management (e.g., weeding of non-target species) was applied in the plots. Each sowing plot consisted of 36 squares of 0.33×0.33 m arranged in a rectangular grid of 1×4 m, which was surrounded by a 0.25 cm disturbed margin in the disturbed plots.

In summer and autumn prior to seed sowing, seeds of species typical of dry grasslands in the region were collected from large populations in grasslands within 5 km from the experimental fields. In 2007 and 2008, 30 and 32 species respectively were collected and sown. Since these two sets of species do not overlap completely, 35 different species were used in the study (Table 1). All seeds were hand cleaned to maximise the number of ripe, viable seeds in the sample. Prior to sowing, seeds were stored in paper bags at room temperature.

**Table 1:** List of all sown species.

	Sown 2007		Established		Flowered		Sown 2008		Established		Flowered	
Species	Sown	Viable	Disturbed	Undisturbed	Disturbed	Undisturbed	Sown	Viable	Disturbed	Undisturbed	Disturbed	Undisturbed
Agrimonia eupatoria*	100	88	9±7.9	6.8±5.3	0	0	100	74	13.1±12.6	3.2±3.5	1.4±2	0
Anthericum ramosum*	100	90	13±6.6	23.2±14.6	0	0	100	94	6.4±7.5	4.4±7.2	0	0
Aster amellus*	100	32	0.3±0.5	2.9±2.8	0	0	100	60	0.7±0.7	2.2±3.2	0	0
*Astragalus cicer*	100	53	1.6±0.8	3.9±2.1	0.1±0.3	0.2±0.6	100	97	1±1.5	1.2±1.6	0	0
*Astragalus glycyphyllos*	-	-	-	-	-	-	100	98	4.4±4.5	5.4±4.7	0.1±0.3	0
Brachypodium pinnatum*	50	45	1.3±1.2	5.1±2.6	0	0	100	93	4.4±2.9	3.3±3.2	0.6±1.1	0
Bromus erectus*	25	21	2.7±1.5	3.6±1.9	0	0	100	42	8.2±6.8	10.9±7.9	1±0.9	0
Bupleurum falcatum*	100	51	2.9±1.9	5.7±3.9	1.6±2.1	0	100	77	6.1±2.8	7.1±6.6	4.4±2.7	0.2±0.4
*Carex flacca*	100	17	0	0.6±0.8	0	0	100	40	0	0	0	0
*Carex tomentosa*	100	20	0	0	0	0	100	22	0	0	0	0
Carlina vulgaris*	100	85	4.4±3.9	9.7±7.6	0.2±0.6	0	100	90	8.6±5.1	7.3±6.5	1.8±2	0
Centaurea jacea*	100	90	5±3.1	10±4.9	2.1±2.2	0	100	88	9.3±4.2	9.4±5.3	5.2±3	0.1±0.3
Centaurea scabiosa*	50	19	1.1±1.4	2.3±2.3	0.4±0.8	0	100	80	6.4±4.1	7.1±6.7	0.3±0.5	0
Coronilla vaginalis*	70	63	1.7±1.5	2.6±1.3	0	0	50	44	1.1±0.7	1±1.4	0.1±0.3	0
*Coronilla varia*	-	-	-	-	-	-	100	95	3.6±2.5	3.4±2.5	0.8±0.8	0.9±1.9
*Filipendula vulgaris*	100	39	0.4±1	4.9±4.6	0	0	100	85	0	0	0	0
*Gentiana cruciata*	-	-	-	-	-	-	100	88	0	0	0	0
*Gobularia elongate*	100	42	0.7±1.1	0.6±1	0	0	100	76	0.1±0.3	0	0	0
*Helianthemum nummularium* subsp. *grandiflorum*	-	-	-	-	-	-	100	69	0	0	0	0
*Inula salicina*	100	24	0	0.1±0.3	0	0	100	82	0	0.1±0.3	0	0
Linum tenuifolium*	100	45	2.7±2.4	3.8±4.2	0	0	100	25	0.7±0.8	0.7±1.2	0.3±0.7	0.3±0.7
*Lotus corniculatus*	100	59	2±1.5	6.3±3.4	0.4±0.7	1.2±1.9	100	94	4.1±3.1	6.4±3.7	3.1±2.4	5.6±4.2
*Odontites lutea*	100	35	0.6±0.8	1.8±3.6	0	0	-	-	-	-	-	-
*Onobrychis viciifolia*	100	56	4.7±3.6	10±4.7	4.2±3.5	5±3.8	100	48	9.2±11.7	12.1±14.8	5.2±6.3	7.3±7.8
Peucedanum cervaria*	100	43	1.3±1.1	3.1±2.9	0	0	100	80	2.7±1.5	3±2.7	0	0
*Primula veris*	-	-	-	-	-	-	100	85	0.4±1.3	0.3±0.7	0	0
*Salvia nemorosa*	100	39	0.8±1.2	1.7±2	0	0	100	38	0.2±0.4	0	0	0
Salvia pratensis*	100	13	0.2±0.4	0.3±0.9	0	0	100	34	5.4±4.9	3.2±2.8	0	0
Salvia verticillata*	100	10	1.7±1.9	0.1±0.3	0.3±0.5	0	100	39	2.6±2.2	0.4±0.7	0.3±0.5	0
Sanguisorba minor*	100	51	4.1±3.6	6±5.1	0.7±0.9	0	100	94	2.3±2.4	4±3.4	0.8±0.9	0
Scabiosa ochroleuca*	100	48	2.1±2.1	2.8±2.9	0.2±0.4	0	100	76	3.9±3.8	2.3±1.9	0.9±1.4	0
*Sesseli hypomarathrum*	100	19	0.6±1.3	0.6±1.1	0	0	-	-	-	-		
*Stachys recta*	100	15	0.1±0.3	0	0	0	-	-	-	-		
Tanacetum corymbosum*	85	20	1.4±1.4	3.1±1.6	0	0	100	66	5.8±4.2	8.2±7.5	0	0
Teucrium chamaedrys*	100	64	2.2±2	5.6±3.4	0	0	100	67	2.1±1.5	2.7±1.3	0	0

Sowing densities in absolute numbers and re-counted for fraction of viable seeds. Mean ± SD of established and flowering individuals is presented for second censuses. Each data point originates from three experimental fields with three sowing plots (replicates) per field. Species marked with asterisks were used in analyses. Nomenclature follows Tutin et al. [70].

Each species was sown in one square that was randomly chosen within the sowing plot; only one species was sown in each square and a few of the squares remained unsown. With a few exceptions (Table 1), 100 seeds per species were sown per square. Seeds were sown by hand on the plot surface. In undisturbed plots, we shook the vegetation and litter in every plot after sowing to dislodge seeds and allow them to come in contact with the soil. In disturbed plots, we gently pressed seeds to the ground to minimize their loss by wind. To assess the mean number of viable seeds sown per species, viability of 3×100 seeds of each species was tested using a 0.1% solution of 2,3,5 - triphenyl tetrazolium chloride ([38]; Table 1).

### Data collection

In September 2008–2010, all sowing plots were carefully examined, and all individuals of each species were counted in their respective squares. The number of flowering individuals was also assessed. To correct for natural regeneration, the mean number of individuals in squares adjacent to the sowing square of a particular species was recorded. The number of naturally recruited individuals was then subtracted from the number of individuals in the sowing square, and the resulting number was used instead. Although many dry grassland species occur in abandoned fields within the study area [32], natural regeneration in the sowing plots was negligible (zero in most species).

Hereafter, we will use the terms first and second census to designate recordings with respect to the year of sowing. The first census of species sown in 2007 was performed in 2008, and the first census of species sown in 2008 was performed in 2009.

For a subset of species sown in 2008 (Table 1), the length of the longest leaf and the number of leaves were measured, since most species develop only earth-bound rosettes in a sterile form. Individual plant size was then assessed as leaf length × leaf number (as an approximation of plant biomass [39]). In most cases, all individuals of a respective species within each sowing square were measured. If a species was abundant, only 20 randomly chosen individuals were measured. Measurements were performed in two consecutive years (2009 and 2010). In the second census (in 2010), however, some plants had already started to flower, and no measurements of plant biomass easily comparable to those carried out for sterile plants were possible due to the different morphology of fertile and sterile plants. Therefore, the maximum size of sterile plants reached by particular species was assigned to each flowering plant for the purpose of our data analyses.

### Data on species traits

We used data collected by D. Průchová (unpubl.) for the plant height and seed weight of all sown species. Plant height was assessed as the height of ten flowering plants randomly sampled in three populations within the study area (30 plants in total). Seed weight was measured for 50 seeds from three populations (150 seeds in total). Seed weight was log10 transformed prior to the analyses. We also used data on the Ellenberg indicator values expressing species requirements for nutrients, light, water, soil reaction, temperature and continentality of each species as species traits [40].

### Data analyses

We analysed data only for 18 species which were sown in both 2007 and 2008 (eight species were not) and were measured for plant size (three species were not). We also omitted species with too low germination rate (less than 1% of seeds germinating) or that were recorded in less than half of the plots (seven species; Table 1). This exclusion was necessary to avoid bias in results because the effects of studied factors were likely to be caused by chance in these sparsely occurring species.

At the first step, we used a generalised linear model (GLM) with a quasi-binomial distribution (a quasi- type distribution was used due to overdispersion of data) to analyse the effects of disturbance, species identity, locality and sowing year as well as their interactions on the number of established individuals. The dependent variable was number of individuals established within one sowing square expressed as the proportion of viable sown seeds of particular species (Table 1; in particular, we used cbind function in R to combine vector of established individuals – successes – with vector of “lost” viable seeds – failures). Due to significant interaction of sowing year with all the three remaining variables (see Table S1 in Supporting Information), we subsequently performed the analyses separately for the two sowing years. Plant size and proportion of flowering individuals were analysed only for species sown in 2008. We also always separately analysed data from the first and second census.

To analyse the effects of disturbance, species identity, locality and their interactions on proportion of flowering individuals, we used a GLM with a quasi-binomial distribution. To analyse the effects of disturbance, species identity, locality and their interactions on plant size, we used a GLM with a Gaussian distribution. Plant size was log_10_ transformed prior to analyses.

To analyse the effects of experimental treatments on plant size, we decided to use the maximum rather than the mean size per plot (i.e., the size of the largest plant of the respective species in the plot). We assumed that mean numbers would be strongly affected by mortality within the plots (small plants dying vs. small plants just surviving). In contrast, the maximum value refers to the potential size the species was able to achieve in a particular plot. Nonetheless, analyses using mean size produced very similar results as analyses with maximum size (data not shown).

To assess the importance of species traits for species-specific responses to disturbance, we performed the same set of analyses as described above (i.e., the analyses of data on the proportion of established individuals, the proportion of flowering individuals and plant size) on data based on sowing in 2008 with species identity being replaced by the value of particular trait of respective species. With this approach, we were able to assess what portion of variability explained by species is directly related to differences in particular traits among species.

We used a quasi-F criterion (ratio of the mean deviances of the explanatory variable and error term) for testing the significance of particular factors and their interactions [41]. This enabled us to take the hierarchical structure of the data into account in the analyses. Species and all interactions with species were tested against residual variability (since each sown seed was replicate for species). Other factors were tested accordingly: disturbance against species × locality (× year), locality against disturbance × species (× year), and year against disturbance × species × locality. In this way, we took into account that disturbance was applied to each species at each locality in each year etc.

The experiment was arranged in blocks. Due to the structure of the data (only 3 blocks in only 3 localities), it was, however, not possible to include block as an additional factor in the models (residual degrees of freedom were too low). The blocks were thus used as a way to arrange the plots, but they were not considered in the tests. Nonetheless, when including block into the model without interactions, the relative importance of particular factors did not change (results not shown) and we can be therefore confident that our results are sufficiently robust. All analyses were performed in R 2.11.0 [42].

## Results

Out of 35 sown species, 32 (nearly 95%) became successfully established in at least some localities or treatments (Table 1). The remaining three completely failed to become established. Seventeen out of the 35 species (almost 50%) started to flower in the second year following sowing, ten of which flowered exclusively in the disturbed plots (Table 1).

### General effect of disturbance and temporal variability

**Figure 1:**
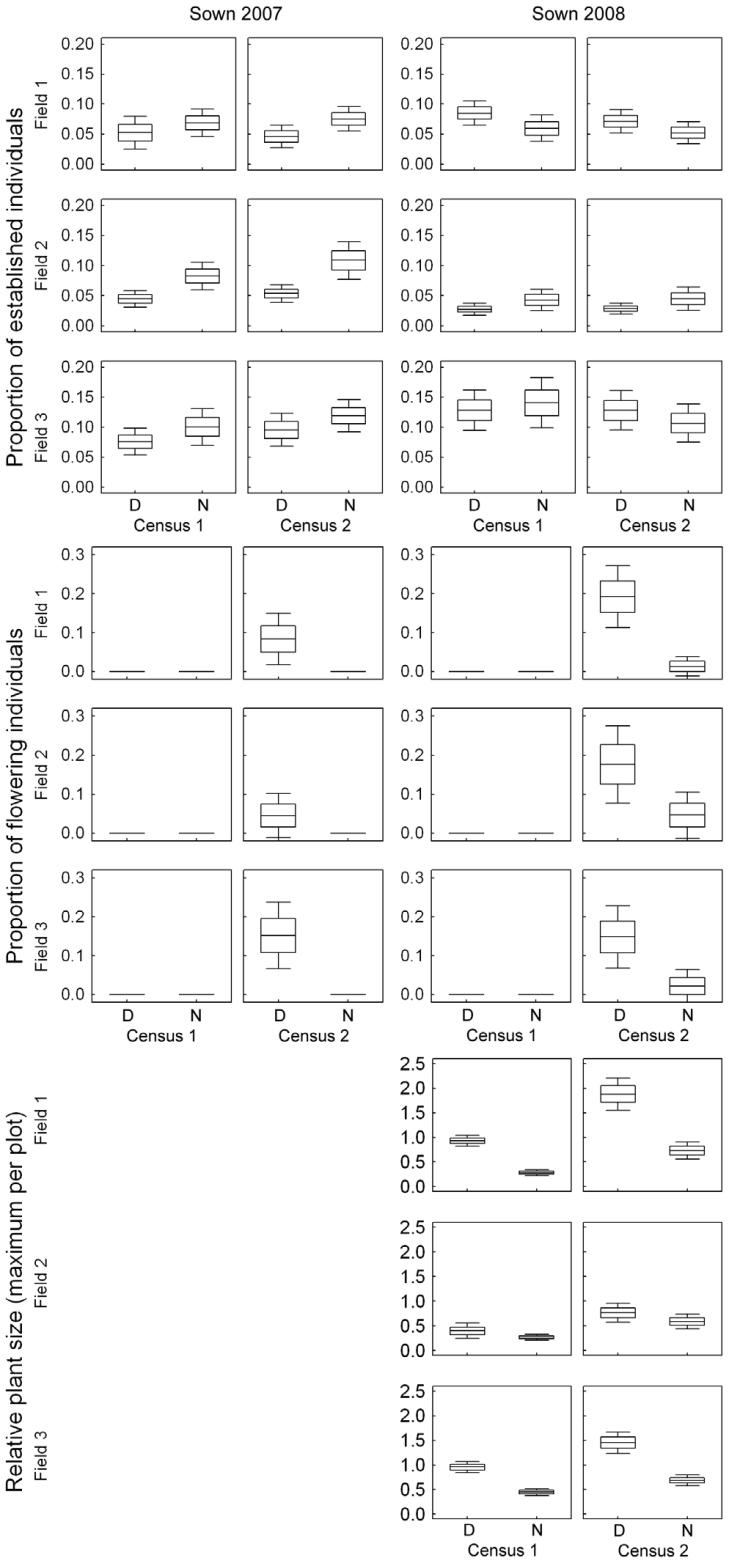
Effect of disturbance on proportion of established individuals, proportion of flowering individuals and relative plant size. For the purpose of graphical presentation only, we need to account for size differences among species. Therefore, relative plant size expressed as ratio between observed plant size (dependent variable in the analyses) and mean size of respective species across all plots and not the real plant size. D – disturbed plots, N – undisturbed plots. Boxes show the mean ± SE, whiskers ±1.96 SE. Census 1 and Census 2 was done in September one and two years respectively following seed sowing. According to soil Phosphorus content and maximum water holding capacity (WHC) we can distinguish the three fields as (1) nutrient rich with low WHC, (2) nutrient poor with low WHC and (3) nutrient poor with high WHC.

Disturbance significantly negatively affected establishment for plants sown in 2007 and the effect was even stronger in the second census. In contrast, no significant effect of disturbance was detected for plants sown in 2008 (Table 2, Figure 1). Disturbance strongly influenced plant size in both censuses, with larger plants growing in the disturbed plots, although the effect was smaller in the second census (Table 2, Figure 1). Even greater positive effects of disturbance were detected in the proportion of flowering individuals (Table 2, Figure 1). The year of sowing interacts with most other factors implying temporal variability in their effects (Table S1).

**Table 2:** Effects of disturbance, species identity, locality and their interactions on proportion of established individuals, proportion of flowering individuals and maximum plant size per plot.

			Census 1			Census 2		
Response variable	Term	Df	R2	Quasi F	*P*	R2	Quasi F	*P*
Established individuals	DISTurbance	1	**0.04**	**9.34**	**0.004**	**0.07**	**16.06**	**<0.001**
sown 2007	SPECies	17	**0.22**	**7.74**	**<0.001**	**0.24**	**8.29**	**<0.001**
	LOCality	2	**0.04**	**4.71**	**0.024**	**0.05**	**7.77**	**0.004**
	DIST×SPEC	17	**0.07**	**2.47**	**0.001**	**0.05**	**1.72**	**0.041**
	DIST×LOC	2	0.01	2.59	0.090	**0.01**	**3.73**	**0.034**
	SPEC×LOC	34	**0.16**	**2.80**	**<0.001**	**0.15**	**2.56**	**<0.001**
	DIST×SPEC×LOC	34	**0.09**	**1.49**	**0.048**	0.07	1.19	0.228
	RESIDUAL	216	0.37			0.36		
								
Established individuals	DISTurbance	1	0.00	0.15	0.704	0.00	0.76	0.390
sown 2008	SPECies	17	**0.34**	**19.93**	**<0.001**	**0.29**	**12.62**	**<0.001**
	LOCality	2	**0.20**	**27.69**	**<0.001**	**0.19**	**28.25**	**<0.001**
	DIST×SPEC	17	**0.06**	**3.56**	**<0.001**	**0.06**	**2.46**	**0.001**
	DIST×LOC	2	0.01	2.85	0.072	0.01	1.70	0.199
	SPEC×LOC	34	**0.14**	**4.14**	**<0.001**	**0.11**	**2.48**	**<0.001**
	DIST×SPEC×LOC	34	0.05	1.47	0.053	0.06	1.28	0.153
	RESIDUAL	216	0.22			0.29		
								
Flowering individuals	DISTurbance	1				**0.36**	**517.47**	**<0.001**
sown 2007	SPECies	17				**0.46**	**20.58**	**<0.001**
	LOCality	2				**0.04**	**842.32**	**<0.001**
	DIST×SPEC	17				0.00	0.00	1.000
	DIST×LOC	2				**0.00**	**547.56**	**<0.001**
	SPEC×LOC	33				0.02	0.53	0.982
	DIST×SPEC×LOC	31				0.00	0.00	1.000
	RESIDUAL	152				0.19		
								
Flowering individuals	DISTurbance	1				**0.36**	**360.98**	**<0.001**
sown 2008	SPECies	17				**0.42**	**14.72**	**<0.001**
	LOCality	2				0.01	2.30	0.131
	DIST×SPEC	17				0.04	1.29	0.208
	DIST×LOC	2				**0.02**	**468.24**	**<0.001**
	SPEC×LOC	33				0.03	0.59	0.961
	DIST×SPEC×LOC	31				0.00	0.10	0.998
	RESIDUAL	152				0.25		
								
Plant size	DISTurbance	1	**0.13**	**65.68**	**<0.001**	**0.10**	**42.09**	**<0.001**
sown 2008	SPECies	16	**0.30**	**14.88**	**<0.001**	**0.34**	**14.16**	**<0.001**
	LOCality	2	**0.09**	**18.47**	**<0.001**	**0.03**	**7.48**	**<0.001**
	DIST×SPEC	17	**0.04**	**2.11**	**0.008**	0.04	1.45	0.115
	DIST×LOC	2	**0.07**	**30.51**	**<0.001**	**0.02**	**4.85**	**0.014**
	SPEC×LOC	34	**0.07**	**1.60**	**0.025**	**0.08**	**1.68**	**0.015**
	DIST×SPEC×LOC	34	0.04	0.96	0.542	**0.08**	**1.68**	**0.015**
	RESIDUAL	216	0.26			0.31		

Significant values are in bold.

### Inter-specific variability

Establishment success was strongly influenced by species identity, slightly more in plants sown in 2008 than in 2007 (Table 2). Even greater difference among species appeared in proportion of flowering individuals (Table 1 and Table 2). Contrary to the prevailing pattern, some species (e.g., *Agrimonia eupatoria, Salvia verticillata*) were more abundant in disturbed plots (Table 1), which probably contributed to the significant effect of the disturbance × species interaction on establishment. In contrast, all species consistently grew larger and flowered more in disturbed plots. Nevertheless, plant size was significantly affected by the disturbance × species interaction in first census (Table 2).

None of the investigated plant traits significantly explained species-specific response to disturbance, neither in proportion of established individuals, proportion of flowering individuals, nor in plant size (*P*>0.05 in all cases).

### Spatial variability

The effect of locality on establishment was relatively small in plants sown in 2007 (fewer individuals established in nutrient rich field compared to the two remaining) and considerable in plants sown in 2008 (more individuals established in field with high WHC compared to the two remaining; Table 2, Figure 1). The effect of locality on plant size and on proportion of flowering individuals was relatively low (Table 2, Figure 1). Individuals sown in 2007 flowered more in field with high WHC compared to the two remaining. No difference in flowering rate between localities was apparent for plants sown in 2008, but they grew smaller in nutrient poor field with low WHC.

Generally, the effect of disturbance either on establishment, size or flowering was consistent across the three fields (Figure 1). However, the effect size differs in some cases resulting in a significant albeit weak effect of disturbance × locality interaction (Table 2). Greater difference in establishment between disturbed and undisturbed plots on the two fields with low WHC compared to the remaining one caused a significant disturbance × locality interaction on establishment only in the second census in plants sown in 2007. No plants sown in 2007 flowered in undisturbed plots and flowering rate in disturbed plots differs between the three fields. It was highest in the field with high WHC and lowest in the nutrient poor field with low WHC. For plants sown in 2008, the difference in flowering rate between disturbed and undisturbed plots was slightly higher in nutrient rich field compared to the remaining two. Considering plant size, the significant interaction was given by the fact that there was no difference in plant size among undisturbed plots on different fields whereas plant size differs between the fields on disturbed plots. It was highest in nutrient rich field and lowest in nutrient poor field with low WHC.

## Discussion

The poor dispersal abilities of many grassland species and the disappearance of source populations are thought to hamper the colonisation of new habitats, such as abandoned fields [31], [34]. When dispersal limitation is overcome (e.g., by means of seed sowing), species can be still limited by unsuitable conditions at a site [26], [34]. Our experiment demonstrated that many dry grassland species are in fact able to recruit, grow and even reproduce within two years, when sown in abandoned fields suggesting that these fields should be considered to be suitable habitats for grassland species. However, we also found that conditions for both establishment and further growth are affected by disturbance indicating impact of vegetation on habitat suitability.

Disturbance can create establishment microsites, areas with reduced competition where seeds can germinate and grow [43], and altered availability of resources important for plant growth (e.g. soil nutrients and light levels [43], [44], [45]). Although it was previously suggested that specific species compositions within sowing plots may have a differential effect on seed germination and seedling performance [3], specific composition of the vegetation was not considered in the present study. We assume that species composition of the resident vegetation is not likely to play an important role in this system as resident vegetation was very homogenous and species poor.

A number of studies have revealed higher seedling establishment rates in disturbed plots than under a vegetation canopy (e.g., [46], [47]–[49]). The negative effect of resident vegetation is mainly attributed to the resulting increased competition for light [5], [11], [50], [51]. In contrast, we found more seedlings in our undisturbed plots, suggesting a facilitative effect of vegetation on establishment. This might imply that severity of competition from resident vegetation is lower than abiotic stress in disturbed plots. There are, however, also other possibilities, why germination rate could be lower in disturbed plots. For example, disturbed soil can have altered abundance and composition of mycorrhizal fungi which affect seedling germination and growth [52], [53].

The positive effect of vegetation canopy on germination rate was found only in plants sown in 2007 and germinating in spring 2008, which was drier than normal. In contrast, above average precipitation was recorded in spring 2009 (especially in May; Appendix S1), when the establishment was comparable in disturbed and undisturbed plots (and even higher in disturbed plots in Field 1; Figure 1). It is therefore likely that the effect of vegetation on seedling establishment is related to moisture. This is in agreement with the results of Bakker *et al*. [18], who documented a positive effect of May and July precipitation on seedling survival and differing effects of experimental management treatments depending on weather. Similarly, Dickson and Foster [17] concluded that seedling establishment react on varying light levels differently in dry and wet years. Temporal variability in the effect of disturbance on seedling establishment could be of course a result of other factors than precipitation as we only have two years of observation and the two years differed also in many other aspects. We, however, think that precipitation is the most likely explanation behind temporal variability as soil moisture plays crucial role for community dynamics of dry grassland plants in this dry environment.

Positive effects of moisture on plant establishment in drier spring conditions are also consistent with higher seedling numbers associated with higher WHC for plants sown in 2007. In the same study region, Münzbergová [3] suggested that water availability and soil reaction limit seedling establishment and might be responsible for high β diversity within the studied dry grasslands. In contrast, higher seedling numbers of plants sown in 2008 were negatively influenced by nutrient availability and standing biomass. This agrees with the findings of Janssens *et al.* [54] who found phosphorus to have strong negative effects on plant recruitment and species diversity in grasslands. We are aware that the evidence on the effect of abiotic factors is quite weak because it is based on three experimental sites only. Moreover, the three experimental fields certainly differ in many other characteristics than those under study, although we believe that site productivity (nutrient content) and water availability are among the most important factors affecting vegetation in this system of dry grasslands. Nonetheless, our results primarily imply that habitat characteristics interact with other temporally variable conditions such as weather. In this light, replication of sowing experiments in different years appears to be necessary to make any general conclusions about factors limiting plant establishment, but the use of this type of approach is still surprisingly rare ([22]; but see e.g. [18], [46]).

Suitable conditions for establishment do not necessarily need to be suitable for growth, survival or reproduction [4], [22], [55]. Indeed, resident vegetation significantly constrained the growth and flowering of established plants in our experiment although more individuals generally became established under a vegetation canopy than on bare ground. Moreover, the effect of disturbance on flowering and on plant size was much stronger than on number of established individuals. Release from competition promoted plant growth and accelerated plant maturity. Although the effect of disturbance on plant size and flowering was always positive, there was obvious difference between flowering rate of plants sown in 2007 and 2008: virtually no plants sown in 2007 flowered in undisturbed plots whereas several species sown in 2008 managed to do so. This seasonal variability in flowering rate further strengthens the difference between different measures of colonization success, as the pattern was very different for numbers of established individuals.

The size of sown plants across the fields was uniformly low under the vegetation canopy, suggesting relatively strong competition from established plants. When released from competition in disturbed plots, plants vary in size depending on soil characteristics. In particular, larger plant size was associated with higher P concentrations and higher WHC. This implies that only in the absence of competitors, sown plants were able to benefit from higher phosphorus content and water supply. We can gain two important conclusions from these results. First, vegetation in studied abandoned fields homogenizes habitat conditions and makes fields generally inhospitable for grassland species. Second, with regard to the above discussed spatial variability in establishment, the effect of phosphorus (and likely also the effect of other factors) is not consistent throughout the plant lifecycle.

Several plant traits have been proposed to be connected with enhanced performance under disturbance regimes or in competition of seedlings with established vegetation [5], [56]–[60]. Our failure to find any of the tested plant traits to be related to species response to resident vegetation could be due to relatively small and homogenous group of investigated species. It is also likely that several antagonistic mechanisms neutralize the effects of seed size. Larger seeds provide more reserves when species have to cope with unfavourable conditions, such as in shadow under vegetation canopy [61]. Large seeded species also present a longer germination time [62] and are hence less prone to fail to establish due to temporarily unfavourable conditions. However, at the same time, seed predators often favour larger seeds [63]. Consequently, vegetation can indirectly negatively affect seedling establishment of large seeded species by providing habitat for seed predators [64]. We also expected smaller plants to suffer more from competition of resident vegetation, but plant height did not explain species-specific reaction to disturbance, although larger plants generally performed better regardless of treatment. This is likely due to relatively fast overgrowth of disturbed plots by non-sown resident species during the first year, which prevented smaller plants from benefiting from competition release.

## Conclusions

Our results imply that the opportunity for a species to successfully colonise an abandoned field (i.e., to establish and especially reproduce) depends to a large extent on the availability of open sites. Such open sites might originate e.g. from disturbances by animals [65], [66], from specific site conditions (e.g., on steeper slopes [32]) or from temporal collapse of whole vegetation canopy (e.g., due to drought [27], [67]). However, in case that such open sites are only transient, they need not to be always exploited by colonizing species. We have shown that colonization is more successful when site opening by disturbance coincide with other suitable conditions such as weather or soil characteristics. It is also likely that timing with respect to plant seasonal cycle is equally important [68].

Seasonal variability involved in our study emphasizes the necessity of temporal replication of sowing experiments. Our results also highlight the importance of following the whole plant life cycle when assessing habitat suitability though we can gain completely different patterns in different phases of the cycle. Although this point has been stressed by some authors [24], it has been overlooked even in recent studies (e.g., [31]). Regarding the effect of resident vegetation on seedling establishment and growth, studies assessing habitat suitability by seed sowing should either involve both vegetation removal treatments and untreated, control plots (e.g., [69]), or deliberately cover the widest range of canopy density within the studied habitat. Such an approach could provide novel insights into factors limiting species distribution.

## Supporting Information

Appendix S1 **Weather of sowing years.**
(XLS)

Appendix S2 **Characteristics of experimental fields.**
(DOC)

Table S1 **Full model including interactions with sowing year.**
(DOC)
