# Supplementary material for: Spatio-Temporal Variation in Contrasting Effects of Resident Vegetation on Establishment, Growth and Reproduction of Dry Grassland Plants: Implications for Seed Addition Experiments
Source: PLoS One. 2013 Jun 5;8(6):e65879. doi: 10.1371/journal.pone.0065879 (PMC3673946; doi:10.1371/journal.pone.0065879)
Supplement: Appendix S2 — Characteristics of experimental fields. (DOC) [file pone.0065879.s002.doc]

**Appendix S2. Detailed description of the three experimental fields.**

In the table, mean ± SD of soil parameters are given.

In PCA of soil nutrients, minerals and water holding capacity (see figure), soil parameters were used as species, localities (experimental fields) as independent nominal variables. Data were centered and standardized by species. First axis explained 74.4%, second axis explained 25.6% of variability in data. Effect of field was significant (*P* = 0.002) in MonteCarlo permutation test with 999 permutations and restricted spatial design. Multivariate analysis was performed in Canoco for Windows 4.5 (Ter Braak et Šmilauer 1998).

|  | Field 1 | Field 2 | Field 3 |
| --- | --- | --- | --- |
| Longitude | 14°19'32.651"E | 14°13'26.244"E | 14°13'36.828"E |
| Latitude | 50°31'35.444"N | 50°31'43.733"N | 50°31'39.465"N |
| Dominant species | *Arrhenatherum elatius*  *Dactylis glomerata* | *Daucus carota*  *Cirsium arvense* | *Elytrigia intermedia* |
| Standing biomass [g/m2] | 470 | 250 | 230 |
| Soil water holding capacity (WHC) | 0.29 ± 0.02 | 0.31 ± 0.03 | 0.34 ± 0.03 |
| pH(H2O) | 7.96 ± 0.08 | 7.99 ± 0.06 | 7.82 ± 0.1 |
| pH(KCl) | 7.65 ± 0.06 | 7.59 ± 0.04 | 7.5 ± 0.04 |
| N [%] | 0.12 ± 0.02 | 0.17 ± 0.02 | 0.15 ± 0.02 |
| C-total [%] | 3.68 ± 0.35 | 8.54 ± 0.23 | 7.82 ± 0.22 |
| C-carbon [%] | 2.19 ± 0.1 | 6.62 ± 0.26 | 6.06 ± 0.21 |
| C-organic [%] | 1.49 ± 0.35 | 1.92 ± 0.29 | 1.76 ± 0.25 |
| Ca [mg/1000g] | 23718 ± 3390 | 32079 ± 2526 | 34634 ± 10062 |
| Mg [mg/1000g] | 119 ± 24 | 180 ± 16 | 146 ± 20 |
| K [mg/1000g] | 116 ± 34 | 479 ± 56 | 284 ± 56 |
| P [mg/1000g] | 10.3 ± 2.6 | 6.5 ± 0.9 | 7.3 ± 1.6 |


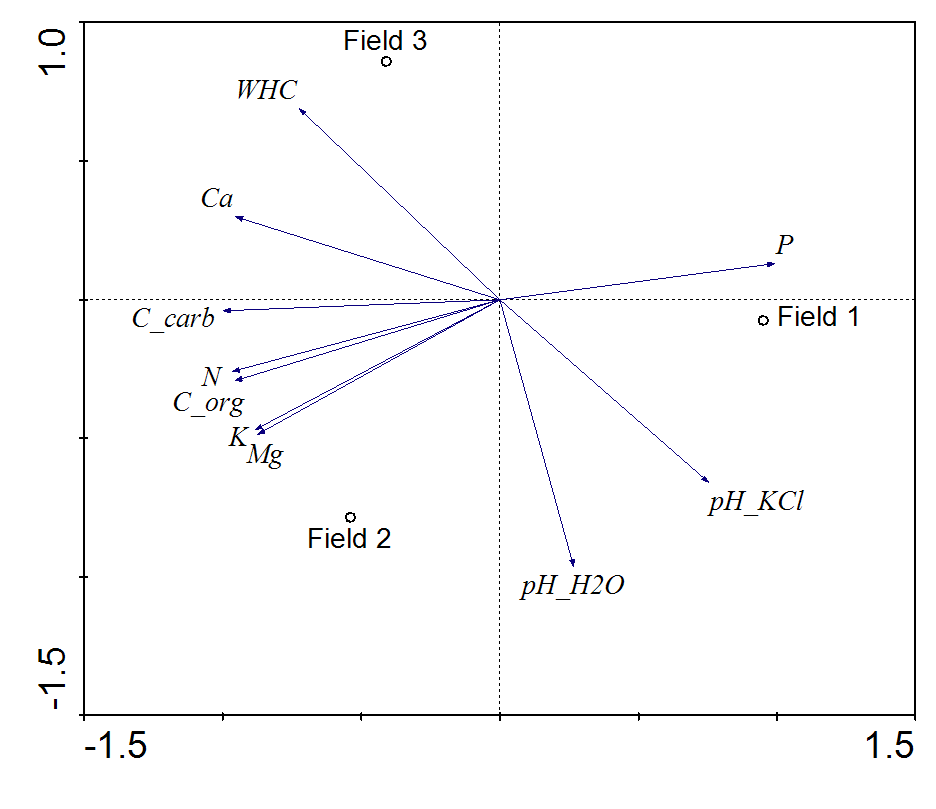


References:

ter Braak, C. J. F., and P. Smilauer. 1998. CANOCO Reference Manual and User's Guide to CANOCO for Windows: Software for Canonical Community Ordination (version 4). Microcomputer Power, Ithaca, New York, USA.
