# Supplementary material for: Spatio-Temporal Variation in Contrasting Effects of Resident Vegetation on Establishment, Growth and Reproduction of Dry Grassland Plants: Implications for Seed Addition Experiments
Source: PLoS One. 2013 Jun 5;8(6):e65879. doi: 10.1371/journal.pone.0065879 (PMC3673946; doi:10.1371/journal.pone.0065879)
Supplement: Table S1 — Full model including interactions with sowing year. (DOC) [file pone.0065879.s003.doc]

Table S1. Effect of disturbance, species identity, sowing year and locality on the proportion of individuals recorded in sowing squares related to the numbers of sown viable seeds. Significant values are in bold.

|  |  |  |  | Census 1 | | |  | Census 2 | | |
| --- | --- | --- | --- | --- | --- | --- | --- | --- | --- | --- |
| Term | Df | Error Term |  | R2 | quasi F | *P* |  | R2 | quasi F | *P* |
| DISTurbance | 1 | SPEC:YEAR:LOC |  | **0.01** | **5.56** | **0.024** |  | **0.01** | **6.41** | **0.016** |
| SPECies | 17 | RESIDUAL |  | **0.22** | **20.98** | **<0.001** |  | **0.19** | **15.02** | **<0.001** |
| Sowing YEAR | 1 | DIST:SPEC:LOC |  | 0.00 | 0.41 | 0.524 |  | **0.01** | **10.06** | **0.003** |
| LOCality | 2 | DIST:SPEC:YEAR |  | **0.09** | **63.74** | **<0.001** |  | **0.08** | **82.48** | **<0.001** |
| DIST:SPEC | 17 | RESIDUAL |  | **0.05** | **4.53** | **<0.001** |  | **0.04** | **3.57** | **<0.001** |
| DIST:YEAR | 1 | DIST:SPEC:YEAR:LOC |  | **0.00** | **8.01** | **0.008** |  | **0.02** | **31.84** | **<0.001** |
| DIST:LOC | 2 | DIST:SPEC:YEAR:LOC |  | 0.00 | 1.98 | 0.153 |  | **0.01** | **5.18** | **0.011** |
| SPEC:YEAR | 17 | RESIDUAL |  | **0.06** | **5.76** | **<0.001** |  | **0.06** | **5.20** | **<0.001** |
| SPEC:LOC | 34 | RESIDUAL |  | **0.09** | **4.28** | **<0.001** |  | **0.09** | **3.42** | **<0.001** |
| YEAR:LOC | 2 | DIST:SPEC:YEAR:LOC |  | **0.03** | **31.62** | **<0.001** |  | **0.03** | **25.86** | **<0.001** |
| DIST:SPEC:YEAR | 17 | RESIDUAL |  | 0.01 | 1.15 | 0.308 |  | 0.01 | 0.66 | 0.840 |
| DIST:SPEC:LOC | 34 | RESIDUAL |  | **0.05** | **2.13** | **<0.001** |  | **0.04** | **1.68** | **0.011** |
| DIST:YEAR:LOC | 2 | DIST:SPEC:YEAR:LOC |  | **0.01** | **7.48** | **0.002** |  | **0.00** | **3.28** | **0.049** |
| SPEC:YEAR:LOC | 34 | RESIDUAL |  | **0.06** | **2.65** | **<0.001** |  | **0.04** | **1.69** | **0.011** |
| DIST:SPEC:YEAR:LOC | 34 | RESIDUAL |  | 0.02 | 0.83 | 0.736 |  | 0.02 | 0.79 | 0.795 |
| RESIDUAL | 432 |  |  | 0.27 |  |  |  | 0.32 |  |  |
